# Evaluating the Effectiveness of Tyrosine Kinase Inhibitors on EGFR Mutations In Vitro

**DOI:** 10.3390/ijms26136157

**Published:** 2025-06-26

**Authors:** Hanshuang Shao, Alan Wells

**Affiliations:** 1 Department of Pathology, University of Pittsburgh, Pittsburgh, PA 15213, USA; has24@pitt.edu; 2 Pittsburgh VA Health System, Pittsburgh, PA 15213, USA

**Keywords:** EGFR, phosphorylation, TKIs, resistance

## Abstract

Abnormal expressions and genetic mutations of EGFR are broadly involved in the progression of many human solid tumors, which has led to the development of small molecule inhibitors (TKIs). However, patients’ tumors usually develop resistance to targeted therapeutic TKIs after a period of treatment, mostly due to secondary mutations in EGFR. To date, three major and prevalent point mutations in EGFR, including L858R, T790M, and C797S, impact the use of TKIs in non-small cell lung cancer patients. Although at least four generations of TKIs have been designed and developed by targeting these mutations, how each mono, dual, or triple variant responds to clinical TKIs remains largely undeciphered. To fill this gap, we constructed a series of EGFR mutants and assessed their responses to clinical TKIs in vitro. The first-generation TKI, erlotinib, completely blocked the autophosphorylation of WT, L858R, C797S, and C797S/L858R, but only partially, if at all, in EGFR containing the T790M mutation alone or in combination. The third generation, osimertinib, completely abolished the autophosphorylation of WT, T790M, L858R, and T790M/L858R. It also significantly inhibited C797S and C790S/L858R, but had no effect on T790M/C797S or T790M/C797S/L858R. EAI045, as the fourth-generation TKI, almost completely inhibited WT and all mutants in complete growth media, but EGF-mediated phosphorylation of WT, C797S, and C797S/L858R were only partially inhibited in quiescence media, while the other mutants were fully inhibited. Furthermore, the abolishment of the enhanced tolerance to Dox in cells transiently expressing T790M/L858R and T790M/C797S/L858R by EAI045 suggests that their enhanced autophosphorylation is involved in their resistant ability. These findings provide some insights into how patients carrying typical mutations should be correctly and efficiently treated and why patients present side effects (because of non-specific inhibitory effects on cells without EGFR mutations).

## Simple Summary

Abnormal expressions and genetic mutations of EGFR are broadly involved in the progression of many human solid tumors, which has led to the development of small molecule inhibitors (TKIs). However, patients’ tumors usually develop resistance to targeted therapeutic TKIs after a period of treatment, mostly due to secondary mutations in EGFR. In this study, we constructed a series of EGFR mutants and assessed their responses to clinical TKIs in vitro. We found that WT and mutant EGFRs responded differently to clinical TKIs. These findings provide some insights into how patients carrying typical mutations should be correctly and efficiently treated and why patients present side effects.

## 1. Introduction

Epidermal growth factor receptor (EGFR), also called ErBb1 or Her1, is a member of EGFR family that includes three additional members, ErBb2, ErBb3, and ErbB4 [1,2,3]. EGFR and its downstream signaling pathways promote cellular proliferation, survival, and migration, all of which are central to the progression of solid tumors [4,5,6,7,8]. Supporting this connection are findings of abnormal expression levels of EGFR and its activation, often by mutation, in many human cancers, including those of the breast, lung, ovary, and colon [3,9,10,11]. Therefore, EGFR activation has been targeted as a therapeutic approach [12]. Unfortunately, EGFR develops acquired resistance to targeted tyrosine kinase inhibitors (TKIs) in many cancer patients due to further mutation(s) [13,14]. To overcome the acquired resistance, newer TKIs have been developed based on the mutant residues and the effect of mutation on the structure of EGFR [12].

The full length of the human EGFR protein consists of 1186 amino acid residues that comprise an extracellular N-terminal ligand-binding domain, a central transmembrane region, an intracellular tyrosine kinase domain, and a C-terminal tyrosine-rich tail [3,15]. The inactive EGFR protein usually exists as monomers on the cell membrane. Once epidermal growth factor (EGF) or another ligand binds to EGFR, it forms homodimers with other EGFR molecules or heterodimers with the rest of the EGFR family members [16]. Consequently, C-terminal key tyrosines, such as Y1068 and Y1173 of one EGFR molecule (called the “receiver” or “receptor”), are phosphorylated by the tyrosine kinase of its partner (called the “activator” or “donor”) [17]. Then, the phosphorylated EGFR triggers its downstream signaling pathways through intermediary kinases, including Akt, MAPK, and STAT3 [18]. Additionally, EGFR can be activated by autophosphorylation in the absence of its ligands [19,20,21]. This phenomenon is very common and often enhanced in many human cancers due to high levels of EGFR or specific missense mutations [22]. For instance, the replacement of the leucine 858 residue with arginine (L858R) leads to ligand-autonomous signaling [3,23]. This mutation, common in non-small cell lung cancers (NSCLCs), led to the development of the first-generation EGFR-targeting TKI erlotinib (brand name: Tarceva) [24,25]. However, it was found that many NSCLC patients developed resistance to erlotinib after about a year of treatment, concurrent with findings of secondary mutations in EGFR [2,26].

To combat tumor resistance, many new TKIs have been developed in recent decades. In NSCLC patients, three prevalent mutants, L858R, T790M, and C797S, occur within the kinase domain of EGFR. As most TKIs function via competing for the ATP binding dock of EGFR with ATP to abolish or reduce its autophosphorylation, structural alterations imparted by these mutations diminish their effectiveness [27,28,29]. Although many TKIs have been developed, the most common TKIs target the clinical EGFR mutants, such as L858R (erlotinib), T790M/L858R (osimertinib), and T790M/C797S/L858R (EAI045) [30,31,32]. Whether and how these TKIs affect the autophosphorylation of wild-type EGFR and EGFR with individual mutations, such as T790M, C797S, T790M/C797S, and C797S/L858R, which may be co-expressed in these tumors, is largely unclear. In this study, we constructed and systematically tested the wild-type EGFR and its mutants, including all three typical mutations (L858R, T790M, and C797S) and related clinical and non-clinical variants through their autophosphorylation and resistance to clinical TKIs. Our findings provide evidence that these individual or dual mutant EGFRs may present differential response patterns from the main clinical mutations. This insight could assist drug developers in designing more specific and efficacious new TKIs against mutated EGFRs that drive cancer progression. 

## 2. Results

### 2.1. C797S Mutation Does Not Enhance Autophosphorylation of T790M/L858R

**Figure 1:**
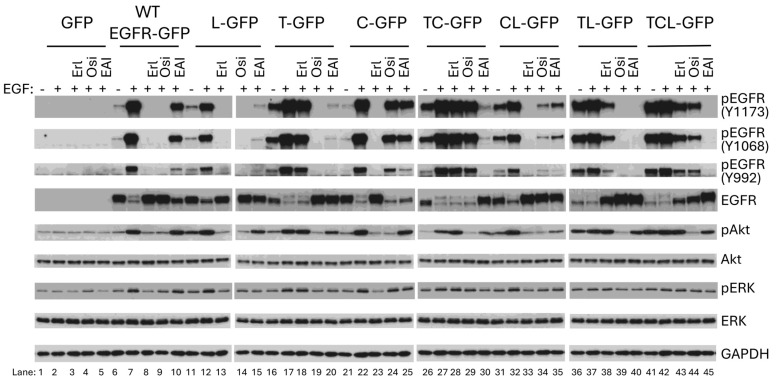
TKI effects on EGFR signaling in WM983B cells. WM983B cells transiently transfected with WT or mutant EGFR tagged with GFP at their C-terminal tail were treated with indicated TKIs at a 10 µM concentration for 30 min prior to being stimulated with 10 nM of EGF for an additional 15 min, followed by extraction for immunoblotting. T = T790M; L = L858R; C = C797S; TL = T790M/L858R; TC = T790M/C797S; CL = C797S/L858R; TCL = T790M/C797S/L858R; Erl = erlotinib; Osi = osimertinib; EAI = EAI045. Representatives of two independent experiments are shown.

Mutations within carcinoma cells lead to quick relapse of lung cancer patients after targeted treatment [13,33,34], with many of these agents targeting EGFR. To improve targeting and overcome the mutational escape of the tumor, three generations of EGFR tyrosine kinase inhibitor have been approved, and a fourth generation is under clinical trial [14,35]. Despite this, the effects of different TKIs on wild-type (WT) and mutant EGFR is not systematically and publicly available. To fill this gap, we constructed a series of EGFR mutants that replicate prevalent clinically identified mutations [29] to test their responses to EGF and TKIs. As we previously reported, we used melanoma cell line WM983B, in which the endogenous EGFR is not detectable at a protein level [3], to transiently express EGFR mutants. This allowed for examination of the mutant EGFR without the background of a ‘wild-type’ EGFR in trans, and for comparison to earlier literature. As shown in Figure 1, all WT and mutant EGFRs were correctly expressed based on the result of immunoblotting (EGFR panels). In line with our previous findings, T790M (pEGFR-Y1173 panels: 16th lane) presented higher levels of autophosphorylation, and T790M/L858R (36th lane) showed the highest autophosphorylation levels in the absence of EGF stimulation compared to WT. Interestingly, the additional C797S mutation that occurred in the T790M/L858R mutant did not significantly enhance the basal autophosphorylation of T790M/C797S/L858R (41st vs. 36th lane), suggesting that its autophosphorylation was almost saturated. Similarly, EGF treatment was also not capable of significantly increasing it (41st vs. 42nd lane), as observed with T790M/L858R (36th vs. 37th lane). EGFR has several key carboxyl tyrosine residues, which tend to be autophosphorylated or phosphorylated upon the binding of its ligands. To determine whether the other key tyrosine residues are also phosphorylated, we performed immunoblotting against pEGFR-Y1068 and pEGFR-Y992 and found that these two tyrosine residues are phosphorylated with a similar pattern with pEGFR-Y1173; the phosphorylation of pEGFR-Y992 was weaker compared to pEGFR-Y1173 and pEGFR-Y1068. This enhancement is also confirmed by the same elevated level of pAkt with and without EGF stimulation (pAkt panels: 41st vs 36th lane; 41st vs. 42nd lane; 36th vs. 37th lane). Due to the enhanced autophosphorylation, we observed a significant degradation in T790M/L858R (EGFR panels: 36th lane) and T790M/C797S/L858R (41st lane), similar to the effect of the EGF treatment for the WT and all other EGFR mutants, further suggesting that both T790M/L858R and T790M/C797S/L858R were almost fully activated. The C797S mutation by itself normally responded to EGF stimulation, as its phosphorylation was largely increased and accompanied by a significant degradation on total EGFR (EGFR panels: 22nd vs 21st lane). Furthermore, C797S did not affect the autophosphorylation of T790M or L858R (pEGFR-Y1173 panels: 26th (TC) vs. 16th (T) lane; 31st (CL) vs. 11th (L) lane). Taken together, these data suggest that all mutants were correctly expressed and normally responded to EGF stimulation, and C797S mutation did not enhance the autophosphorylation of T790M/L858R in EGFR-negative melanoma cells.

### 2.2. EGFR Mutants Respond Differently to Targeted TKIs

TKIs targeting specific EGFR mutations have been developed for at least four generations because NSCLC patients quickly become resistant to TKIs due to the acquisition of additional mutation(s) [14]. Next, we were interested in examining the responses of EGFR mutants, including mono, dual, and triple mutations, even though some mutations have not been reported to naturally occur in patients. Understanding their real responses to TKIs may be beneficial to the design and development of new TKIs with more specificity and potency. To perform this examination, we used three generations of TKIs, including erlotinib, osimertinib, and EAI045 to treat cells. 

We found that erlotinib completely blocked the EGF-mediated phosphorylation of WT, L858R, C797S, and C797S/L858R. It partially inhibited T790M/L858R and T790M/C797S/L858R but had no effect on T790M and T790M/C797S. The fact that erlotinib completely blocked C797S and C797S/L858R suggests that replacing C797 with S does not affect the activated tyrosine kinase activity of EGFR caused by the R858 mutation. 

Osimertinib completely inhibited the EGF-mediated phosphorylation of WT, L858R, T790M, and T790M/L858R. It significantly inhibited C797S/L858R but had little effect on C797S and T790M/C797S/L858R. T790M/C797S was completely resistant to osimertinib inhibition. EAI045 completely blocked the EGF-mediated phosphorylation of T790M/L858R and T790M/C797S/L858R. It slightly inhibited WT and C797S and significantly inhibited L858R, T790M, and C797S but not completely. Notably, EGF-mediated phosphorylation resulted in the dramatic degradation of total EGFR. In line with the inhibitory content of EGF-mediated phosphorylation by tyrosine kinase inhibitors, the total EGFRs increased correspondingly. 

In GFP-expressing cells, we did not observe any enhanced phosphorylation of Akt and ERK when EGF was applied. The levels of both pAkt and pERK were significantly boosted when cells expressing WT, L858R, T790M, C797S, T790M/C797S and C797S/L858R were treated with EGF. In line with the highest autophosphorylation, T790M/L858R and T790M/L858R did not present enhanced pAkt and pERK when the cells were treated with EGF. Mirroring its effect on EGFR autophosphorylation, erlotinib completely blocked pAkt and pERK in EGF-treated WM983B cells expressing WT, L858R, C797S, and C797S/L858R. It did not affect pAkt and pERK in every T790M-containing mutant EGFR. The complete inhibition of EGF-mediated phosphorylation of the WT indicates the non-specific effect of erlotinib on normal tissues in which wild-type EGFR is expressed when applied in cancer patients. 

Osimertinib completely blocked EGF-mediated pAkt and pERK of WT, L858R, T790M. In T790M/L858R- and T790M/C797S/L858R-expressing cells, osimertinib restored EGF and EGFR autophosphorylation-mediated pAkt to the parental WM983B level. For C797S and T790M/C797S, osimertinib just slightly affected their EGF-mediated phosphorylation but downregulated pAkt to the non-EGF treatment basal level, suggesting that osimertinib binds to other upstream signaling molecules of Akt but not ERK prior to the addition of EGF. 

EAI045 did not affect EGF-mediated pAkt in WT-expressing WM983B cells, suggesting that there is no side effect on normal tissue and organ in cancer patients. EAI045 also did not inhibit EGF-stimulated pAkt in C797S-expressing cells. It significantly blocked pAkt in WM983 cells expressing L858R, T790M, T790M/C797S, and C797S/L858R. Surprisingly, EAI045 completely blocked EGF-mediated phosphorylation of T790M/L848R and T790M/C797S/L858R, but just slightly decreased pAkt levels, suggesting that these two mutants may enhance pAkt independently of the phosphorylation of EGFR at tyrosine 1173, at least in these melanoma cells.

### 2.3. Nutritional Quiescence Stabilizes Akt

**Figure 2:**
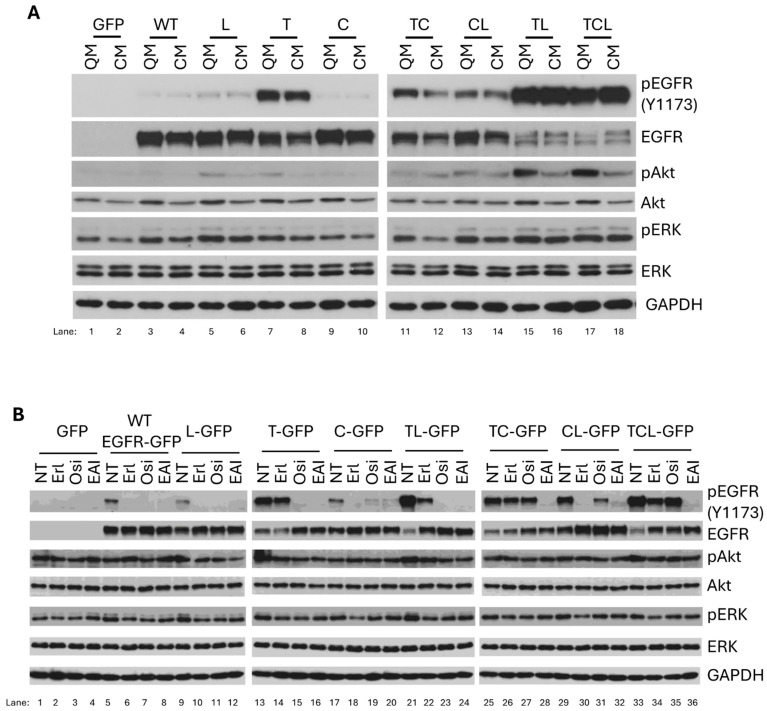
Autophosphorylation of EGFR in WM983B cells. (**A**) Immunoblotting of indicated proteins from WM983B cells transiently expressing WT or EGFR mutants grown in quiescent or complete growth media. All WT and mutant EGFRs were tagged with GFP. QM stands for quiescence media; CM stands for complete media (see Section 2 for composition). (**B**) WM983B cells were transiently transfected with WT or mutant EGFRs for 4 h and then switched to complete media for culture overnight. After treatment with 10 µM of indicated TKI for 30 min, cells were collected for immunoblotting against indicated proteins. T = T790M; L = L858R; C = C797S; TL = T790M/L858R; TC = T790M/C797S; CL = C797S/L858R; TCL = T790M/C797S/L858R. Erl = erlotinib; Osi = osimertinib; EAI = EAI045. Representatives of two independent experiments are shown.

To mimic the physiological condition, we next used complete cell growth media to determine the autophosphorylation of EGFR mutants in WM983B cells. First, we compared the autophosphorylation of WT and mutant EGFR in quiescent media to complete growth media. As shown in Figure 2A, WT, L858R, and C797S demonstrated very low autophosphorylation in both quiescent and complete media. The autophosphorylation of T790M, T790M/C797S, and C797S/L858R was significantly elevated compared to that of WT, L858R, and C797S. Again, T790M/L858R and T790M/C797S/L858R presented the highest autophosphorylation levels. Interestingly, we noted that the level of autophosphorylation of both T790M/L858R and T790M/C797S/L858R were very similar in quiescent and complete media, but the pAkt level in complete media was significantly downregulated compared to quiescent media. This is likely due to the decreased amount of total Akt in complete growth media caused by activation-mediated Akt degradation. However, pERK does not present similar significant differences in both media. As WT and EGFR mutants demonstrate similar autophosphorylation in quiescent and complete media, other factor(s) or signaling pathway(s) must be involved in the enhanced degradation of EGFR mutants, especially for T790M/L858R and T790M/C797S/L858R in complete growth media. 

For this reason, we were interested in determining the effects of three generations of TKIs on EGFR mutants in complete growth media. Like in quiescent media, we observed the same inhibitory effects of erlotinib on WT, L858R, C797S, and C797S/L858R in complete media (Figure 2B). It partially inhibited T790M/L858R and T790M/C797S/L858R but did not affect T790M and T790M/C797S. Osimertinib completely blocked the autophosphorylation of WT, L858R, T790M, and T790M/L858R and dramatically inhibited C797S and C797S/L858R but had no effect on T790M/C797S and T790M/C797S/L858R. To our surprise, the autophosphorylation of WT and C797S was almost completely inhibited by EAI045 in complete growth media, while it abolished the autophosphorylation of other mutants, suggesting that EAI045 has a broader range of activities in complete media. Furthermore, osimertinib just downregulated the enhanced phosphorylation of Akt in T790M/L858R- and T790M/C797S/L858R-expressing cells to the basal level, similar to cells with GFP, WT, and other mutants. EAI045 also reverted the enhanced pAkt in T790M/L858R- and T790M/C797S/L858R-expressing cells to the basal level. To determine whether the responses of the wild-type and mutant EGFRs to TKIs were not cell type-dependent, we performed the same tests in other cell lines, including melanoma cell line WM983A (Figure S1A), breast cancer cell line MCF-7 (Figure S1B), and human non-small cell lung carcinoma cell line H1299 (Figure S1C). We found that the patterns of their autophosphorylations and responses to TKIs in these three cell lines were very similar to that in the WM983B cell line, suggesting that these TKIs work on wild-type and mutant EGFRs in a cell-independent manner. Taken together, these results suggest that osimertinib and EAI045 work differently in quiescent and complete media, and therefore, ex vivo testing is needed to validate the in vivo situation. 

### 2.4. Mutations Confer Resistance to General Chemotherapies

These mutations in EGFR are considered to drive oncogenic progression and tumor aggressiveness [36], and they are, therefore, targeted. However, as the targeted biologics are given in combination with standard chemotherapy, we queried whether cells expressing different EGFRs responded similarly to each other when challenged by chemotherapy.

**Figure 3:**
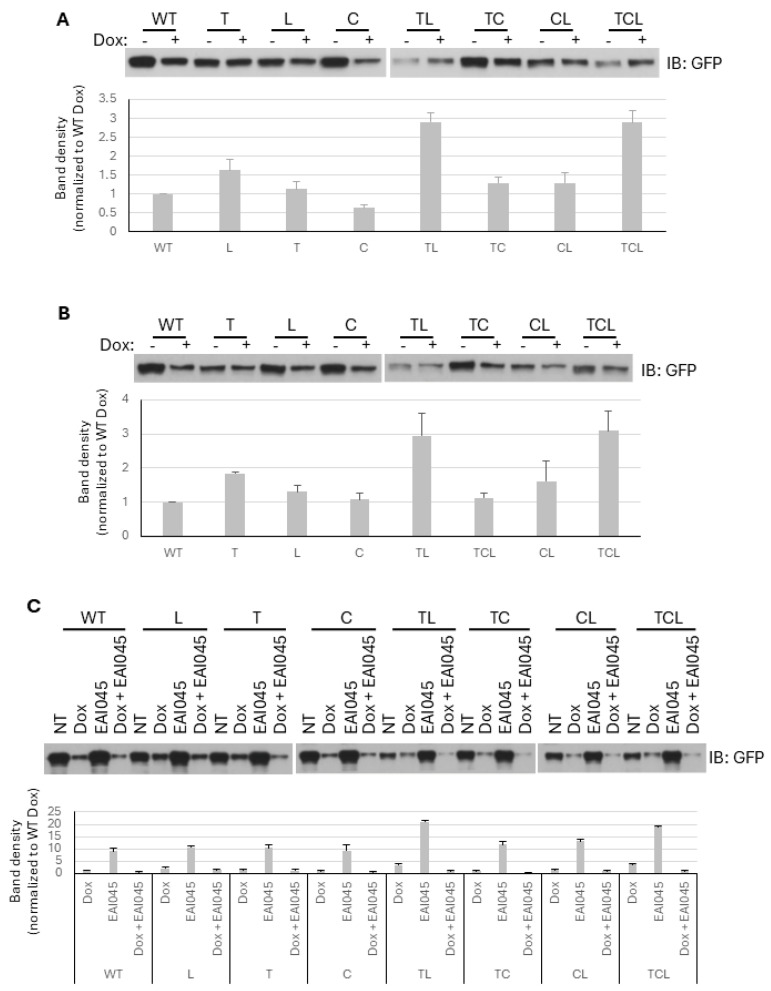
Resistance of EGFR-expressing melanoma cells to doxorubicin. (**A**,**B**) WM983B (**A**) and WM983A (**B**) cells were transfected transiently with GFP-tagged WT or mutant EGFR for 4 h and switched to growth media for overnight culture. Two (**A**) or twelve (**B**) micromolars of Dox was applied to cells for a further 24 h (**A**) or 48 h (**B**) of incubation. Adherent cells were extracted with 2× SDS sample buffer in the presence of β-mercaptoethanol for immunoblotting against GFP. The extraction buffer volumes were 200 µL and 100 µL for the NT and Dox-treated cells, respectively. A twenty micromolar of each sample was loaded on gel for immunoblotting. (**C**) WM983B cells were pretreated with 10 µM of EAI045 for 30 min prior to adding 2 µM of Dox for further culture. The density of each band in A, B, and C was measured using Image J 1.54 software and normalized to WT NT, as shown in the graphs. T = T790M; L = L858R; C = C797S; TL = T790M/L858R; TC = T790M/C797S; CL = C797S/L858R; TCL = T790M/C797S/L858R. Erl = erlotinib; Osi = osimertinib; EAI = EAI045. Data are mean ± SD of three independent experiments.

We chose the generalized cytotoxic doxorubicin (Adriamycin) (Dox), as it kills above 80% of the melanoma WM983B and WM983A cells at different concentrations after one or two days, respectively, according to our previous findings [3]. Since the stable expression of T790M/C797S/L858R could not be achieved in either WM983A or WM983B cells, immunoblotting against GFP-tagged WT or mutant EGFR was performed on adherent cells after Dox treatment to determine the relative resistance of EGFR mutants to Dox. This was carried out to normalize for cells that retained expression of the EGFR molecules. As shown in Figure 3A,B, cells expressing EGFR that contained L858R or T790M mutation demonstrated resistance to Dox, with both WM983B and WM983A cell lines behaving similarly. Of interest, when the EGFR contained both of these mutations, the level of resistance to killing by Dox appeared synergistic and not just additive. The C797S mutation did not lead to any increase in cell survival, nor did it appear to contribute to the resistance conferred by the other mutations. To further confirm that the enhanced resistance to Dox, especially for T790M/L858R and T790M/C797S/L858R, was due to their hyper-autophosphorylation, we pretreated the WM983B cells after transient transfection with EAI045 for 30min prior to adding Dox for further incubation. As shown in Figure 3C, EAI045 significantly decreased the survival of all WT and mutant EGFRs expressing WM983B cells, suggesting that the autophosphorylation levels of EGFR directly contributed to the resistance to Dox.

## 3. Discussion

After efficacy, the side effects of chemotherapy in cancer patients are an important point that drug developers need to consider. Epoch-making progress occurred when targeted chemotherapeutic inhibitors were designed and developed. For example, erlotinib, the first TKI for NSCLC patients who carry the L858R mutation in EGFR, dramatically inhibited the proliferation of cancer cells, thereby shrinking tumors and extending the lives of patients when concurrently used with chemotherapeutic drugs [37]. However, the side effects of erlotinib were broadly seen in many patients, suggesting that it also inhibited the function of WT EGFR at least partially, especially in the skin and intestine, where EGFR is highly expressed and involved in homeostatic tissue turnover [38,39]. This unfortunate and therapeutically limiting side effect may be inherent in EGFR-targeting TKIs, as all three generations of TKIs have significantly or completely inhibited the autophosphorylation of WT EGFR in both melanoma WM983B (Figure 1) and WM983A (Figure S1A), breast cancer MCF-7 (Figure S1B), and human non-small cell lung carcinoma H1299 (Figure S1C) cell lines cultured in complete growth media, which most likely mimic human physiological conditions. This was not due to the cell type, as all three cells presented a similar pattern. This suggests that all three generations of TKIs we tested in this study also bind to WT WGFR in addition to mutant EGFRs. For EGF-mediated phosphorylation of WT EGFR in quiescence media, both erlotinib and osimertinib also completely inhibited the strong EGF-mediated phosphorylation of WT in both WM983B and WM983A cells. EAI045 also presented significant, though not complete, inhibition, further suggesting that all three TKIs would negatively affect normal tissue function. Furthermore, we found that osimertinib presented significant cellular toxicity (as shown in Figure S2, 10 µM of osimertinib dramatically killed all the cell lines tested, including the melanoma cell lines, the breast cancer line MB-MDA-231, and even fibroblasts). In contrast, the same concentrations of erlotinib and EAI045 did not affect the growth of those cell lines. Interestingly, 10 µM of osimertinib had no effect on the inhibition of the growth of MCF-7, suggesting that the lethal role of osimertinib was not due to its purity. One possible reason for the toxicity of osimertinib might be its high dosage. Therefore, we investigated whether we could determine a concentration of osimertinib that would completely block the autophosphorylation of T790M/L858R but had no effect on WT EGFR. Indeed, as shown in Figure S3, the autophosphorylation of T790M/L858R was almost completely inhibited by 0.3 µM of osimertinib but had no effect on WT EGFR. We then determined the effect of 1 µM of osimertinib on cell growth and found that this concentration did not affect the cell growth of WM983A, WM983B, or A549 (Figure S4). Wu et al. also reported that a moderate concentration of osimertinib presented some effect on WT EGFR [40]. These findings and our present study also suggest that osimertinib can be used at a low concentration at the beginning of clinical treatment to minimize its side effects. Then, the dose can be increased after several months of treatment or whenever osimertinib resistance appears to block WT EGFR in intercellular transferred exosomes, even though a higher concentration of osimertinib will result in some amount of side effects on normal tissue. Furthermore, we found that EAI045 also presented a potent effect on the inhibition of all EGFR mutants tested in the present study, as well as WT, when a 10 µM concentration was applied (Figure S5A). However, when its concentration was reduced to 1 µM, it only completely blocked the autophosphorylation of L858R, T790M/L858R, and T790M/C797S/L858R but had no effect on WT (Figure S5B). The rest of the mutants were also significantly inhibited by 1 µM of EAI045. Even though EAI045 has not moved forward to clinical trials for unknown reasons, our findings here, revealing its potent inhibition of mutations but not WT at a low concentration, might provide some insight for the design of next-generation TKIs that will maintain the inhibitory potence of EAI045 but with high stability and strong intracranial function.

Although the development of TKIs has had huge impacts on disease, TKI resistance is still a challenge for cancer patients and drug developers. In addition to EGFR mutagenesis, an important factor of EGFR-TKI susceptibility is the allelic status of C797S and T790M mutations. When C797S and T790M are localized in *trans*, combining the first- and third-generation EGFR-TKIs can be effective for EGFR/T90M/C797S. When both of these mutations are present in *cis*, then resistance to the first and the second generations emerges, and applying orthoallosteric fourth-generation EGFR-TKIs may be effective [41,42]. For this reason, we constructed our combination mutant EGFR on the same molecule to represent *cis* mutants. Furthermore, intercellular transfer of exosomal WT EGFR also triggered osimertinib resistance in NSCLC [40]. 

Erlotinib was developed to target EGFR presenting the L858R mutation to block its enhanced autophosphorylation, even though the autophosphorylation level of L858R is less than that of WT or EGFR with the T790M mutation (Figure 2B). As expected, erlotinib completely inhibited both the autophosphorylation and EGF-mediated phosphorylation of L858R but had no effect on T790M. Interestingly, it also significantly inhibited T790M/L858R and partially inhibited T790M/C797S/L858R in complete growth media and quiescence media in the presence of EGF. If the T790M mutation was a consequence of erlotinib-mediated mutation in NSCLC patients under treatment, the autophosphorylation of T790M/L858R might not be inhibited by erlotinib at all. Therefore, a possible explanation is that some T790M/L858R-containing NSCLC cells already existed before erlotinib treatment, but the population was very small. This is in line with Fujiwara’s findings [43]. Contrarily, osimertinib did not affect the triply mutated EGFR T790M/C797S/L858R; this suggests that the C797S mutation was very possibly selected due to pressure from osimertinib. These findings suggest that a clinical retrial of an earlier targeted TKI may be warranted, as tumors progressively accumulate mutations to abrogate current therapies.

These studies were performed in melanoma cell lines rather than lung cancer cell lines, for which anti-EGFR TKIs are FDA approved. This was done for several reasons. First, the proliferation of melanoma cell lines in vitro is sensitive to EGFR kinase blockade [44]. Second, the melanoma cell lines used allowed us to distinguish between effects on localized cancer (WM983A—radial growth) and invasive cancer (WM983B—vertical growth) from the same patient. Lastly, these cell lines express only very low levels of ErbB2 and no detectable amounts of Axl [45], the two most common transphosphorylation partners of EGFR. This allows for a more straightforward analysis of receptor activation and signaling. The cross-phosphorylation and activation of other receptors with tyrosine kinase activity can promulgate signaling secondary to EGF binding to EGFR, such as we have reported for Tyro3 [46], but this is mainly seen in addition to EGFR auto-phosphorylation. Even if the interactions are not at the autophosphorylation site, such as for ACTN4 with EGFR [47], the signaling through such pathways is reflected by autophosphorylation. Thus, the autophosphorylation detection herein serves as a marker of activation rather than a specific site of downstream signaling. A more vexing possibility is kinase-independent signaling, which we have reported substantially in earlier work [48], but this was noted only when its binding partner with kinase activity, ErbB2, was present at high levels (as the transphosphorylation was inefficient in the absence of EGFR kinase activity). 

The ability of wild-type and mutant EGFR molecules to interact with each other was reported by Zhu et al. [49] in vitro using computer simulation. Nukaga et al. [50] also reported that the amplification of wild-type EGFR alleles occurred in non-small cell lung cancer cells, resulting in resistance to drugs. This finding suggests that the heterodimer of wild-type and mutant EGFR molecules might be formed in non-small cell lung cancer cells, thereby affecting the binding of selective tyrosine kinase inhibitors. Such a situation could impact the efficacy of the inhibitors or the resistance of the cells. The melanoma cell lines WM983A and WM983B used in the present study did not express detectable EGFR and only very low levels of Axl [another partner for EGFR heterodimers], in order to cleanly dissect the specific effects of the inhibitors on the individual mutant EGFR. Therefore, when these studies are extended to human lung cancer cell lines, the expression levels of EGFR, HER2, HER3, and Axl need to be determined and accounted for. We also need to distinguish the expression levels of wild-type and mutant EGFR in a certain lung cancer cell or cell line if they are co-expressed. Whether HER2, HER3, and Axl are involved in the enhanced autophosphorylation of EGFR mutants, such as T790M/L858R and T790M/C797S/L858R in lung cancer cells, relevant siRNAs will be used to downregulate their expression and then re-expression of wild-type or mutant EGFR-GFP, respectively or the co-expression of wild-type and mutant EGFR-GFP. Still, given these limitations of the current short communication, future work is directed at expanding these findings to other cancers, primarily NSCLC, in both in vitro and in in vivo model systems, and determining the network of critical downstream signaling elements, including *trans*-activated receptor kinase family members.

## 4. Materials and Methods

### 4.1. Reagents and Cell Culture

Polyclonal antibodies, including EGFR, pEGFR(Y1173), pEGFR(Y1068), pEGFR(Y992), pAkt(S473), Pan-Akt, pERK, ERK, and GAPDH, were purchased from Cell Signaling Technology (Danvers, MA, USA). Monoclonal GFP antibody was purchased from Santa Cruz Biotechnology (Dallas, TA, USA). Lipofectamine 2000 was purchased from Life Technologies (Grand Island, NY, USA). Tyrosine kinase inhibitors erlotinib, osimertinib, and EAI045 were purchased from MilliporeSigma (Burlington, MA, USA). Melanoma cell lines WM983B and WM983A were cultured in a complete growth medium consisting of 3 parts DMEM (1 gL^−1^ glucose) and 1 part Leibovitz’s L-15 medium with 1× Pen/Strep antibiotics and 10% fetal bovine serum (CM: complete media). Human breast cancer cells MCF-7 were cultured in RPMI 1640 medium consisting of 10% fetal bovine serum and 1 × Pen/Strep antibiotics. Human non-small cell lung carcinoma cell line H1299 was cultured in DMEM medium with the addition of 10% fetal bovine serum, 1x Pen/Strep antibiotics, and 1 g/L glucose.

### 4.2. Mutagenesis 

The generation of mutant EGFR C797S, T790M/C797S, C797S/L858R, and T790M/C797S/L858R was performed using oligo primer-guided polymerase chain reaction (PCR). All combination mutational constructs were constructed on the same molecule and should be considered ‘*cis*’ for translation into their in vivo counterparts. The PCR products were purified, digested, and cloned into expression vector pEGFP-N1 to construct C-terminal GFP-tagged EGFR; the pEGFP-N1 vector was also used as the ‘negative’ control constructs. All positive colonies were identified by restriction enzyme digestion. The mutations were further confirmed by Sanger DNA sequencing. 

### 4.3. Transfection

Melanoma WM983A and WM983B cells were briefly treated with trypsin and then seeded at an appropriate concentration in a 12-well plate containing 1 mL of complete growth medium and cultured overnight to reach about 60% cell confluency at transfection. The next day, to form a complex of Lipofectamine and DNA, 5 µL of Lipofectamine 2000 and 2 µg of plasmid DNA were diluted in 125 µL of Opti-medium with low serum. After incubating at room temperature for 5min, two dilutions were gently mixed and incubated at room temperature for 30 min. During incubation, the overnight culture media was replaced with fresh media containing only 0.1% dialyzed fetal bovine serum (QM: quiescent media). Finally, the mixture was slowly added to each well and incubated overnight or for 4 h, followed by replacing the transfection media with fresh complete growth media until treatment with inhibitors.

### 4.4. Immunoblotting

After transfection and treatment, adherent cells were gently rinsed with phosphate-buffered saline (PBS) solution, followed by lysing in RIPA buffer containing 1× protease inhibitors cocktails set V (Temecula, CA, USA). The samples were then transferred to microtubes and incubated on ice for 5 min prior to brief sonication. After centrifugation at 13,000× g at 4 °C for 30 min, the supernatant of each sample was carefully transfected to a new tube, and the total protein concentration was determined using a BCA™ Protein Assay (Thermo Scientific™ Pierce™, Rockland, IL, USA). After adding a one-fifth volume of 5× sodium dodecyl sulfate (SDS) sample buffer containing β-mercaptoethanol, the samples were incubated in a boiling water bath for 5min, and then 10 µg of total protein from each sample was loaded onto an appropriate concentration of acrylamide gel based on the size of the target protein. After gel running, the proteins were transferred to a polyvinylidene difluoride (PVDF) membrane made of acrylamide gel. Then, the PVDF membrane was incubated in 5% fat-free milk dissolved in 1 × TBS buffer with 0.1% Tween-20 (TBST) at room temperature for a half hour, followed by further incubation with the appropriately diluted primary antibody under constant slow rotation at 4 °C overnight. The next day, the membrane was washed with 1 × TBST 4 times for 5 min each time and incubated with the secondary antibody with appropriate dilution at room temperature for 45 min. Finally, the membrane was thoroughly washed and developed using ECL reagents and a processing machine.

### 4.5. Cell Death Assay

The cells were transfected transiently with WT or mutant EGFR-GFP overnight, followed by incubation with the indicated concentration of Dox for 24 h for the WM983B cells and 48 h for the WM983A cells. Then, the adherent cells were rinsed with PBS to remove nonadherent and poorly adherent cells (indicative of dead cells) and extracted by an appropriate volume of 1 × SDS sample buffer containing β-mercaptoethanol. Finally, the amount of EGFR-GFP protein in the non-treatment (NT) and Dox-treated groups was determined using immunoblotting against GFP antibody. For the Dox and EAI045 co-treatment, the transiently transfected cells were pre-incubated with EAI045 for 30 min prior to adding the Dox.

## 5. Conclusions

Herein, we report that cells expressing EGFR with single and multiple mutations present different sensitivities to newer-generation TKIs, and that the efficacy of such agents against the various EGFR mutants is not predictable. Our study demonstrates that the C797S mutation does not enhance the autophosphorylation of EGFR mutant T790M/L858R, but it significantly reduces the sensitivity of T790M/L858R to the third-generation TKI osimertinib. Although clinical TKIs are suggested to be specific to EGFR mutants, this study addresses the nonspecific inhibitions of clinical TKIs on WT EGFR when a high concentration applied. These findings provide some insights into how patients carrying typical mutations could be most efficiently treated, why patients present side effects due to non-specific inhibitory effects of high concentrations of osimertinib on cells without EGFR mutations, and how to design new TKIs for cancers carrying triply mutated T790M/C797S/L858R EGFR. 

## Data Availability

All relevant data for this study are presented herein and in the Supplementary Materials. Additional details are available on request from the corresponding author.

## Supplementary Materials

The following supporting information can be downloaded at: https://www.mdpi.com/article/10.3390/ijms26136157/s1.
