# Supplementary material for: Evaluating the Effectiveness of Tyrosine Kinase Inhibitors on EGFR Mutations In Vitro
Source: Int J Mol Sci. 2025 Jun 26;26(13):6157. doi: 10.3390/ijms26136157 (PMC12249685; doi:10.3390/ijms26136157)
Supplement: Supplementary file 1 [file ijms-26-06157-s001.zip › ijms-3575431-supplementary.pdf]

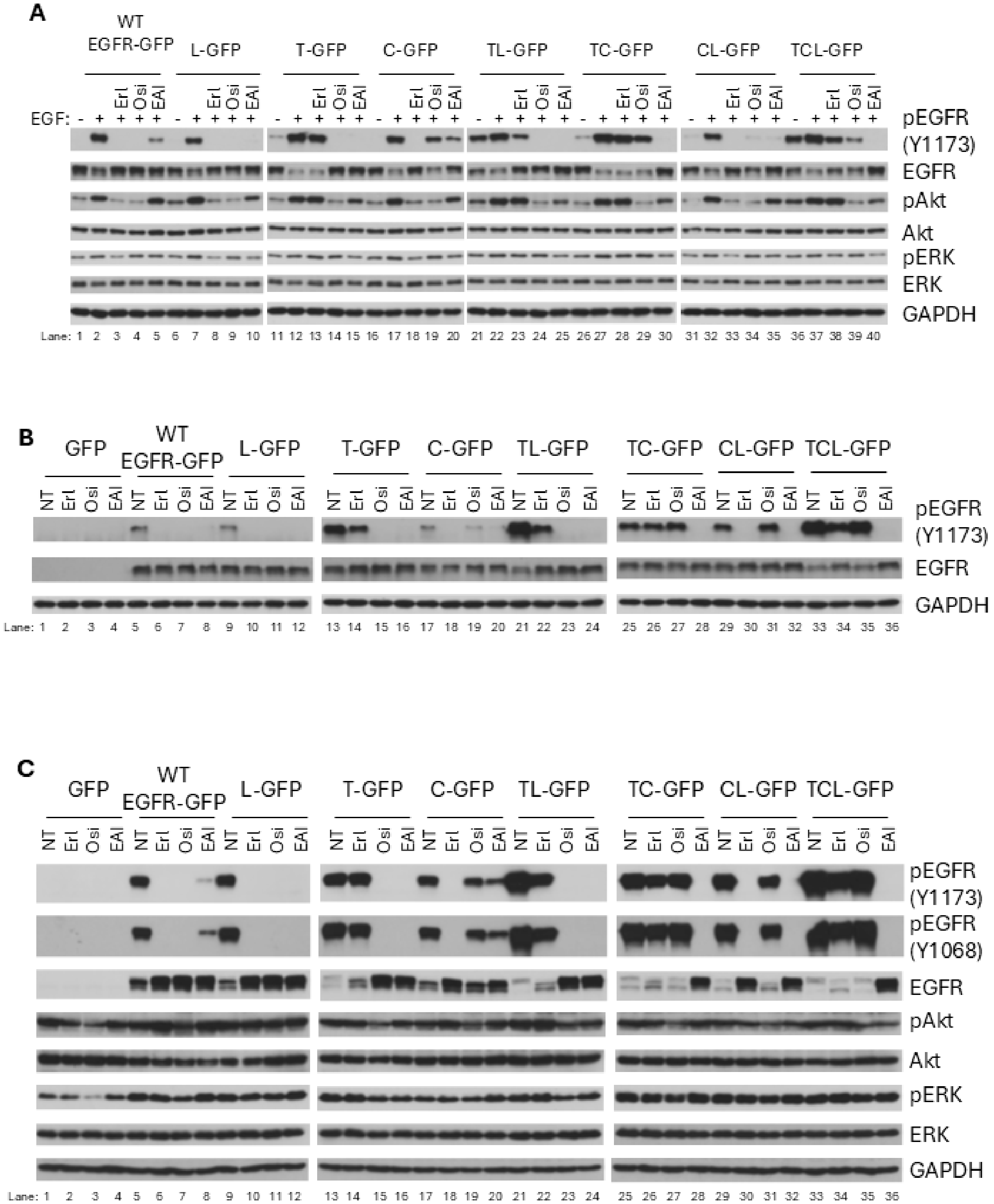


Figure S1. Phosphorylation of EGFR mutants in WM983A, MCF-7 and H1299 cells. (A) Immunoblottings of indicated proteins from WM983A cells transiently expressing WT or EGFR mutants grown in quiescent media treated with 10 nM EGF for 15min in the absence or presence of 10 µM of indicated TKIs pretreatment. (B, C) Immunoblottings of indicated proteins from MCF-7 (B) and H1299 (C) cells transiently transfected with WT or mutant EGFR and treated with 10µM indicated TKI for 30min in growth media. Shown are representatives of two independent experiments.

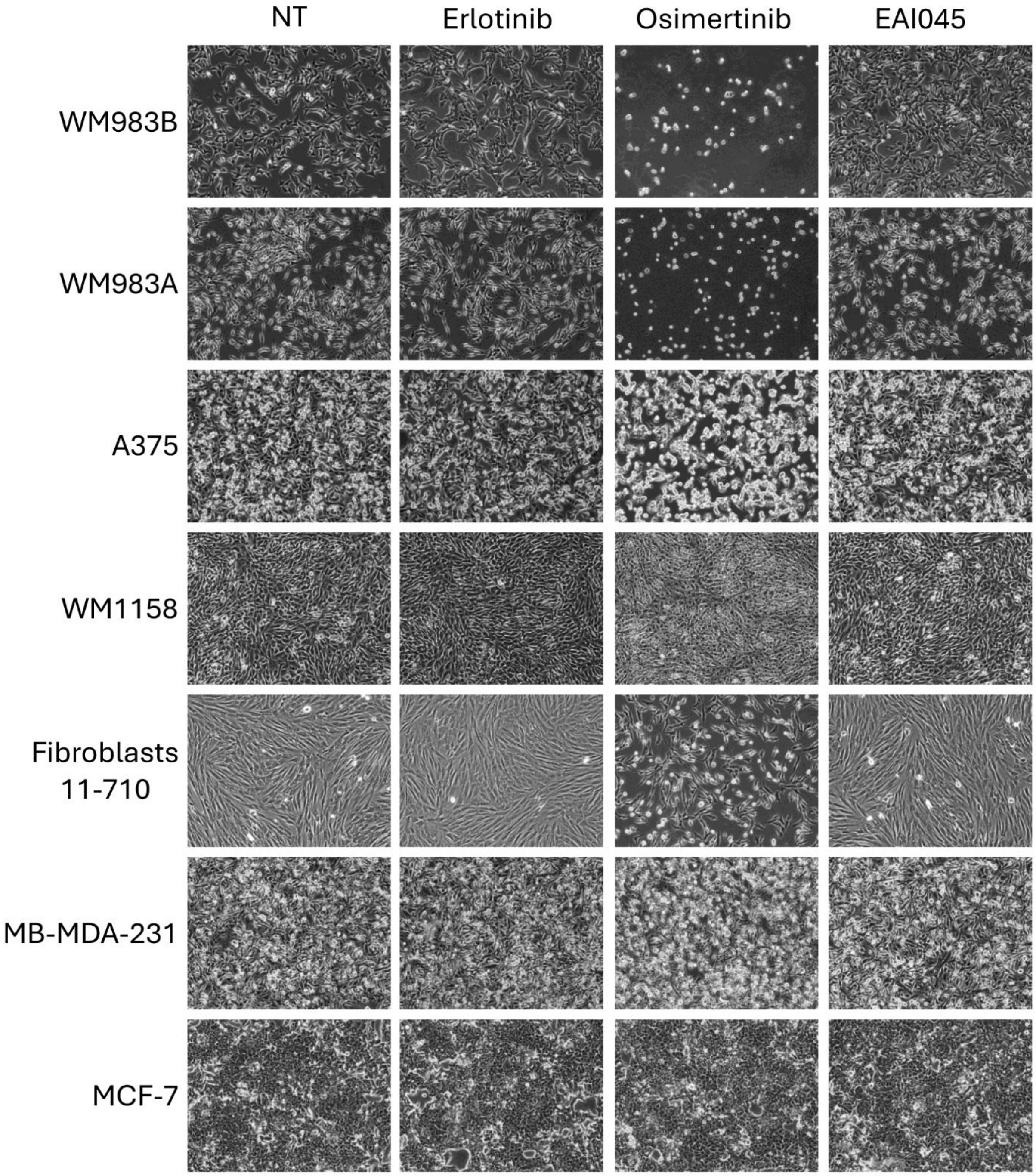


Figure S2. Effect of Osimertinib on the growth of cells. The indicated cells were seeded in 6-well plates in complete growth media for 16h to get about 50% confluency the next day. Then, cells were cultured in growth media with 10 μM of indicated TKIs for 24h. Images were randomly taken under a microscope. Shown are representatives of two independent experiments.

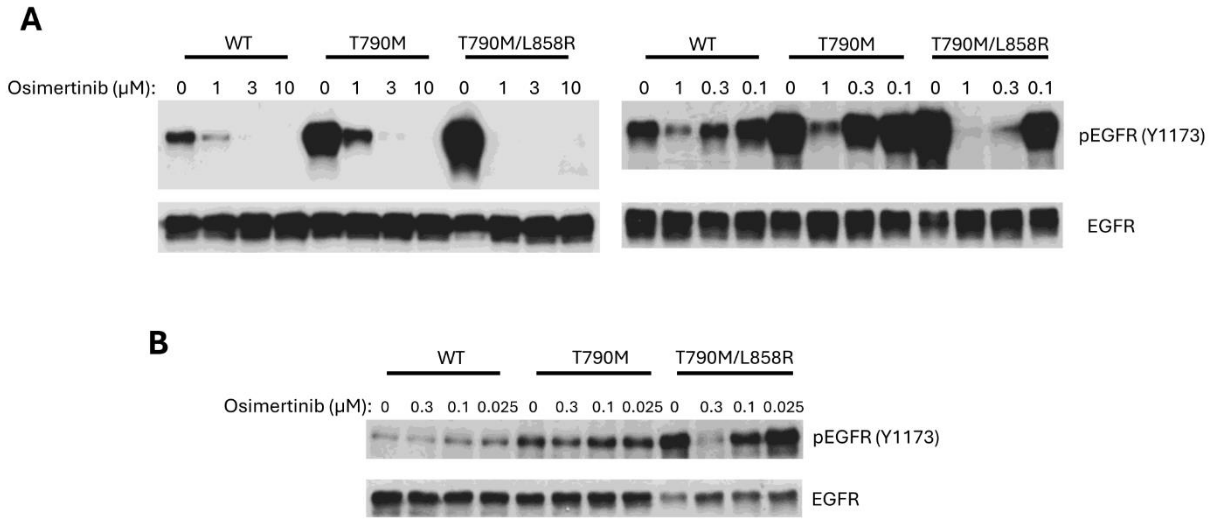


Figure S3. Phosphorylation of EGFR mutants in H1299 cells. (A) Immunoblottings of indicated proteins from H1299 cells transiently expressing WT, T790M and T790M/L858R grown in growth media in the presence of indicated concentration of Osimertinib for 30 min. (B) Immunoblottings of indicated proteins from stable H1299 cells treated with indicated concentration of Osimertinib for 30 min in growth media. Shown are representatives of two independent experiments.

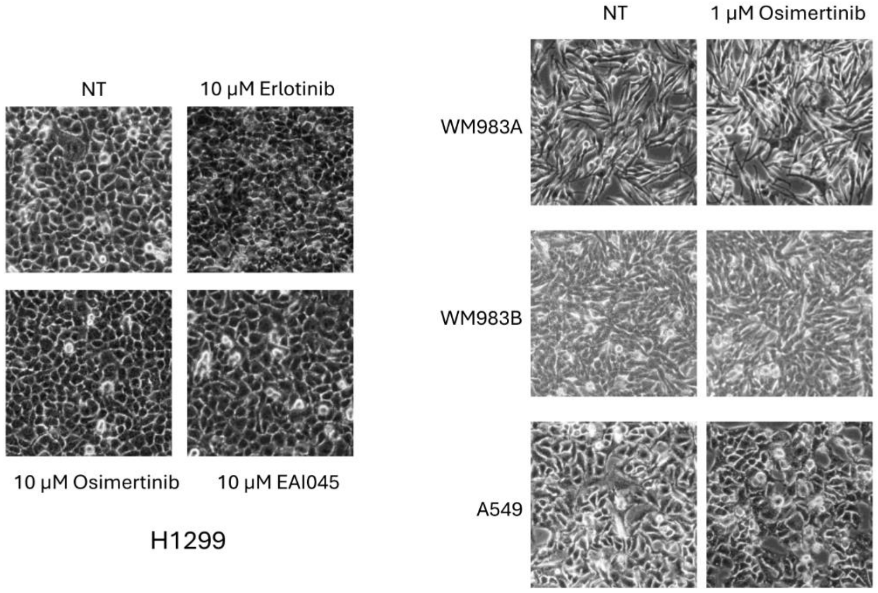


Figure S4. Effect of TKIs on the growth of cells. The indicated cells were seeded in 6-well plates in growth media for 16h to get about 50% confluency the next day. Then, cells were cultured in growth media with 10 µM of indicated TKIs (A) or 1 µM of Osimertinib (B) for 24h. Images were randomly taken under a microscope. Shown are representatives of two independent experiments.

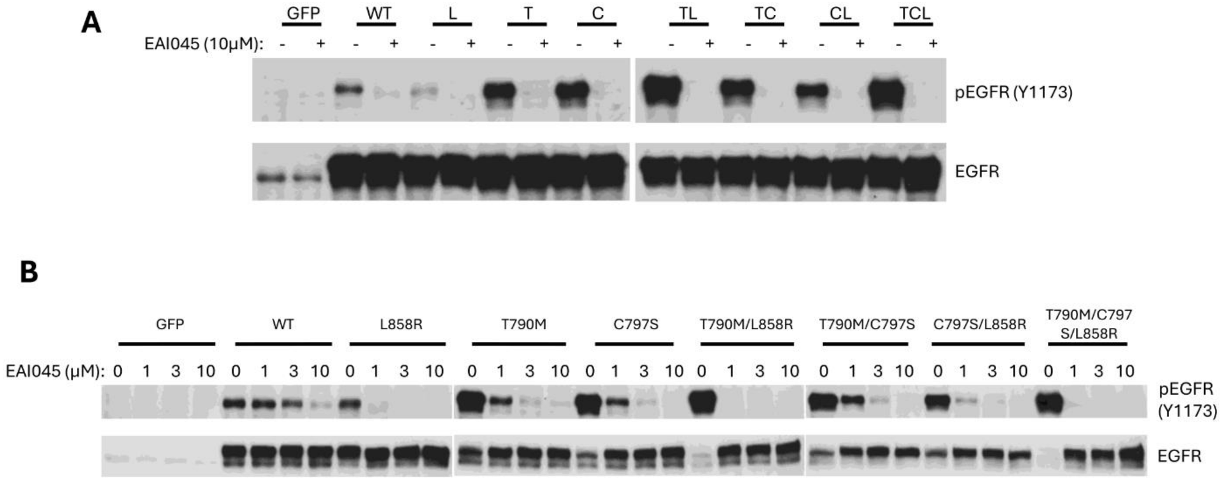


Figure S5. Phosphorylation of EGFR mutants in EAI045 treated stable H1299 cells. Immunoblottings of indicated proteins from stable H1299 cells treated with 10 μM (A) or indicated concentration (B) of EAI045 for 30 min. Shown are representatives of two independent experiments.
